# Signalling mechanisms that regulate metabolic profile and autophagy of acute myeloid leukaemia cells

**DOI:** 10.1111/jcmm.13737

**Published:** 2018-08-17

**Authors:** Olga Pereira, Alexandra Teixeira, Belém Sampaio‐Marques, Isabel Castro, Henrique Girão, Paula Ludovico

**Affiliations:** ^1^ Life and Health Sciences Research Institute (ICVS) School of Medicine University of Minho Braga Portugal; ^2^ ICVS/3B's ‐ PT Government Associate Laboratory Braga/Guimarães Portugal; ^3^ Institute for Biomedical Imaging and Life Science (IBILI) Faculty of Medicine University of Coimbra Coimbra Portugal

**Keywords:** acute myeloid leukaemia, autophagy, energetic metabolism, glycolysis, mitochondrial oxidative phosphorylation, nutrient‐sensing pathways

## Abstract

Acute myeloid leukaemia (AML) comprises a heterogeneous group of hematologic neoplasms characterized by diverse combinations of genetic, phenotypic and clinical features representing a major challenge for the development of targeted therapies. Metabolic reprogramming, mainly driven by deregulation of the nutrient‐sensing pathways as AMPK, mTOR and PI3K/AKT, has been associated with cancer cells, including AML cells, survival and proliferation. Nevertheless, the role of these metabolic adaptations on the AML pathogenesis is still controversial. In this work, the metabolic status and the respective metabolic networks operating in different AML cells (NB‐4, HL‐60 and KG‐1) and their impact on autophagy and survival was characterized. Data show that whereas KG‐1 cells exhibited preferential mitochondrial oxidative phosphorylation metabolism with constitutive co‐activation of AMPK and mTORC1 associated with increased autophagy, NB‐4 and HL‐60 cells displayed a dependent glycolytic profile mainly associated with AKT/mTORC1 activation and low autophagy flux. Inhibition of AKT is disclosed as a promising therapeutical target in some scenarios while inhibition of AMPK and mTORC1 has no major impact on KG‐1 cells’ survival. The results highlight an exclusive metabolic profile for each tested AML cells and its impact on determination of the anti‐leukaemia efficacy and on personalized combinatory therapy with conventional and targeted agents.

## 1 INTRODUCTION

Acute myeloid leukaemia (AML) comprises a group of heterogeneous hematopoietic disorders characterized by a multitude of genetic/epigenetic aberrations, altered differentiation, proliferation and self‐renewal of hematopoietic stem cells and myeloid progenitors.^1^, ^2^, ^3^ AML intensive chemotherapy regimens have favourable outcomes in young patients^4^ but limited application and poor outcomes among elderly, the most affected population.^5^, ^6^, ^7^ Given the genetic, phenotypic and clinical diversity among the AML patients, the development of targeted therapies remains a major challenge.^8^ Therefore, the elucidation of the mechanisms underlying the multi‐stages and multi‐causal pathogenesis of AML is demanding.

A switch from mitochondrial oxidative phosphorylation (OXPHOS) to glycolytic metabolism, recognized as “Warburg effect,” is a common strategy used by cancer cells to overcome their bioenergetics needs.^9^, ^10^ This metabolic reprogramming provides tumour cells with advantages necessary for sustaining their high proliferation rates, such as the rapid generation of ATP and intermediates for the synthesis of fatty acids, nucleotides and amino acids.^11^ Studies in AML cell lines and human primary AML blasts correlated metabolic reprogramming with chemo‐resistance showing that enhanced glycolysis decreases the AML cells sensitivity to cytarabine, while the inhibition of glycolysis potentiates the cytotoxicity of this anti‐leukaemia agent.^12^ Furthermore, it was also proposed that the extent of myeloblast glycolysis may be an effective method to determine the pretreatment prognosis of AML.^13^

Importantly, the metabolic reprogramming in cancer cells is mainly associated with the deregulation of the major nutrient‐sensing pathways: the AMP‐activated protein kinase (AMPK), the mammalian target of rapamycin complex 1 (mTORC1) and the phosphoinositide 3‐kinase (PI3K)/serine/threonine protein kinase B (AKT).^14^ Deregulation of these signalling pathways, which enhance cellular survival and proliferation, seems to cooperate with genetic abnormalities to the pathogenesis of AML.^15^ In fact, while PI3K/AKT pathway is often found activated in AML, mTORC1 appears to be active in all reported AML cases.^16^, ^17^ Both mTORC1 and AKT seem to contribute for the glycolytic metabolism of some AML cells and human primary AML blasts.^18^, ^19^ Globally, it is still debatable and controversial if the deregulation of AMPK, mTORC1 and/or AKT in AML cells would function as a tumour suppressor or promoter.^15^, ^17^, ^20^, ^21^, ^22^, ^23^, ^24^, ^25^, ^26^ Nevertheless, once activated, AMPK^27^, ^28^, ^29^ and AKT^29^, ^30^ may control macroautophagy in mTORC1‐(in)dependent pathway(s). Macroautophagy, hereafter referred as to autophagy, is a multi‐step self‐degradative process by which cytoplasmic content, such as long‐lived proteins and superfluous/damaged organelles, is delivered to lysosomes for degradation.^31^ Deregulation of autophagy has been extensively described in AML acting both as tumour promoting and suppressing.^26^, ^32^, ^33^, ^34^ Therefore, the elucidation of the interconnection between the nutrient‐sensing players, autophagy and energetic metabolism is of major relevance to understand cellular homeostasis and survival of AML cells. Herein, results provide evidence that different AML cells present diverse metabolic profiles. Indeed, whereas KG‐1 cells exhibited preferential OXPHOS metabolism with co‐activation of AMPK and mTORC1 associated with increased autophagy flux, NB‐4 and HL‐60 cells displayed high intracellular ATP levels and a glycolytic profile mainly associated with AKT/mTORC1 activation and low autophagy flux. Inhibition of AKT is disclosed as a promising target for therapeutic intervention in some scenarios while inhibition of AMPK and mTORC1 has no major impact on KG‐1 cells survival.

## 2 MATERIAL AND METHODS

### 2.1 Cell culture

The NB‐4, HL‐60 and KG‐1 cell lines were obtained from the German Collection of Microorganisms and Cell cultures (DSMZ^®^ ‐ Deutsche Sammlung von Mikroorganismen und Zellkulturen—German). The cells were maintained in RPMI 1640 medium (Biochrom^®^) supplemented with 10% heat‐inactivated fetal bovine serum (FBS; Biochrom^®^) and 1% antibiotic‐antimycotic solution (Invitrogen^®^) in a humidified, 37°C, 5% CO_2_ atmosphere.

### 2.2 Treatments

Compound C (CC) was purchased from Sigma‐Aldrich^®^ and dissolved in dH_2_O. Rapamycin (Rap) and bafilomycin A1 were also obtained from Sigma‐Aldrich^®^ but dissolved in DMSO. MK‐2206 was purchased from Bertin Pharma^®^ and prepared in DMSO. Final concentration of compounds: CC—2.5 μmol/L; Rap—2 μmol/L, bafilomycin A1—10 nmol/L; MK‐2206—20 μmol/L.

NB‐4 and HL‐60 cells were submitted to MK‐2206, while KG‐1 cells were exposed to CC or Rap. All tested AML cells were treated with bafilomycin A1 for the assessment of autophagy flux. To study the glycolytic metabolism dependence, AML cells were cultured for 24 hours in RPMI 1640 medium depleted of glucose (Alfagene^®^) supplemented with 10% heat‐inactivated FBS (Biochrom^®^), 1% antibiotic‐antimycotic solution (Invitrogen^®^) and 11 mmol/L 2‐Deoxy‐D‐glucose (2‐DG; Sigma‐Aldrich^®^) in a humidified, 37°C, 5% CO_2_ atmosphere.

### 2.3 Determination of the extracellular glucose and lactate levels

NB‐4, HL‐60 and KG‐1 cells were plated at 0.5 × 10^6^ cells/mL/well, cultured for 24 hours with or without the respective treatment(s), collected and the supernatant reserved. Measurement of the extracellular glucose and lactate levels was performed using the glucose test kit from R‐Biopharm^®^ and the lactate test kit from Spinreact^®^ according to the manufacturer's instructions. At least, 3 independent biological replicates were performed.

### 2.4 Quantification of the intracellular ATP levels

NB‐4, HL‐60 and KG‐1 cells were plated at 0.5 × 10^6^ cells/mL/well, cultured for 24 hours, collected and the pellet reserved. Intracellular ATP levels were determined using the ENLITEN ATP Assay System from Promega^®^ according to the manufacturer's instructions. At least, 3 independent biological replicates were performed.

### 2.5 Measurement of cell viability—Annexin V/PI assay

NB‐4, HL‐60 and KG‐1 cells were plated at 0.5 × 10^6^ cells/mL/well, cultured for 24 hours with or without the respective treatment(s) and collected. The cells were then washed with 800 μL of phosphate‐buffered saline (PBS) followed by the addition of 100 μL of binding buffer to each sample. An incubation with 5 μL of annexin V (BD Biosciences^®^) and 10 μL of propidium iodide (PI) at 50 μg/mL (Invitrogen^®^) was then performed for 15 minutes at room temperature in the dark. Two hundred microlitres of binding buffer was added once again to each sample. PI signal was measured using the FACS LSRII flow cytometer (BD Biosciences^®^) with a 488‐nm excitation laser. The annexin V signal was collected through a 488‐nm blocking filter, a 550‐nm long‐pass dichroic with a 525‐nm band pass. Signals from 10 000 cells/sample were captured, and FACS Diva was used as the acquisition software. Analysis of the results was performed using the FlowJo 7.6 (Tree Star^®^) software. At least, 3 independent biological replicates were performed.

### 2.6 Immunoblotting analysis

Protein extraction from NB‐4, HL‐60 and KG‐1 cells upon 24 hours of culture with or without the respective treatment(s) (0.5 × 10^6^ cells/mL/well were plated) was performed with 100 μL of lysis buffer (1% NP‐40; 500 mmol/L Tris‐HCL, 2.5 mol/L NaCl, 20 mmol/L EDTA, phosphatase and protease inhibitors (Roche^®^); pH 7.2). Twenty micrograms of the total protein was resolved in a 12% sodium dodecyl sulphate (SDS) polyacrylamide gel and transferred to a nitrocellulose membrane for 7 or 12 minutes in the Trans‐Blot Turbo Transfer System (Bio‐Rad^®^). Membranes were blocked for 1 hour in tris‐buffered saline (TBS) with 0.1% Tween‐20 (TBS‐T) containing 5% bovine serum albumin (BSA; Sigma‐Aldrich^®^) and afterwards incubated overnight at 4°C with the polyclonal primary antibodies at 1:1000 in 1% BSA—rabbit anti‐phospho‐AMPKα (Thr172) antibody; rabbit anti‐AMPKα antibody; rabbit anti‐phospho‐ACC (Ser79) antibody; rabbit anti‐ACC antibody; rabbit anti‐phospho mTORC1 (Ser2448) antibody; rabbit anti‐mTORC1 antibody; rabbit anti‐phospho‐p70 S6K (Thr389) antibody; rabbit anti‐p70 S6K antibody; rabbit anti‐phospho‐AKT (Ser473) antibody; rabbit anti‐AKT antibody; rabbit anti‐LC3A/B antibody; rabbit anti‐GAPDH antibody (all from Cell Signaling Technology^®^) and mouse anti‐actin antibody (Abcam^®^). After washing with TBS‐T, membranes were incubated with the secondary IgG anti‐Rabbit antibody (Cell Signaling Technology^®^), at 1:5000 in 1% skim milk for 1 hour 20 minutes at room temperature. Protein levels were detected after incubation with Clarity Western ECL Substrate (Bio‐Rad^®^) or SuperSignal West Femto Maximum Sensitivity Substrate (Thermo Scientific^®^). Digital images were obtained in the ChemiDoc XRS System (Bio‐Rad^®^) with the Quantity One software (Bio‐Rad^®^). At least, 3 independent biological replicates were performed.

### 2.7 Immunofluorescence assay

After 24 hours of culture, NB‐4, HL‐60 and KG‐1 cells (50 000 cells/plate) were re‐suspended in PBS and fixed in a slide using the cytospin technique. Fixation was then performed in 2% paraformaldehyde (PFA). Cells were washed, permeabilized and blocked with 4% BSA in PBS 0.05% Tween. Incubation with primary antibody, rabbit anti‐mouse LC3 A/B (Cell‐Signaling^®^), was performed overnight at 4°C. Goat anti‐rabbit IgG Alexa Fluor 588—red‐fluorescent dye—(Molecular Probes^®^) was used for 1 hour as secondary antibody. Cells were also stained with DAPI (4′,6‐diamidino‐2‐phenylindole) that binds to DNA regions, marking the cell nuclei. An epifluorescence microscope (BX61 microscope with an Olympus DP70 camera) was used to slide visualization, and images were analysed with ImageJ^®^ Software (National Institutes of Health). At least, 3 independent biological replicates were performed.

### 2.8 Statistical analysis

All data are reported as the mean ± standard error of the mean (SEM). Statistical analysis was performed using the 2‐away ANOVA and Bonferroni's post hoc tests to denote significant differences between the tested groups for the annexin V/PI approach. Student's *t* test was applied to compare the extracellular glucose and lactate levels between untreated and MK‐2206 treated HL‐60 and NB‐4 cells. The one‐way ANOVA and Tukey's post hoc tests were used to compare the tested groups for all the other approaches. A *P*‐value lower than 0.05 was assumed to denote a significant difference.

## 3 RESULTS

### 3.1 Glycolytic versus oxidative metabolism of AML cells

**Figure 1:**
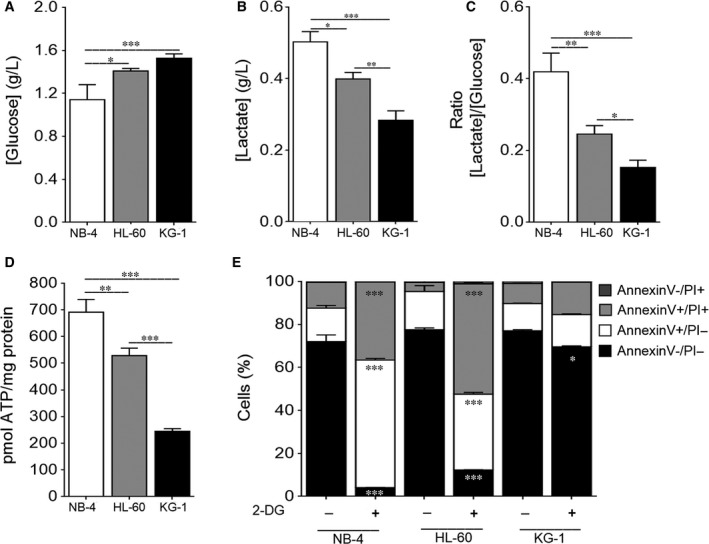
NB‐4 cells present a high glycolytic metabolism followed by HL‐60 and KG‐1 cells. NB‐4, HL‐60 and KG‐1 cells were maintained for 24 h in normal growth medium. (A) Extracellular glucose and (B) lactate levels were determined using glucose and lactate enzymatic detection kits. (C) The ratio between the extracellular lactate and glucose levels ([Lactate]/[Glucose]) was calculated. (D) Intracellular ATP levels were assessed by the ENLITEN ATP Assay System. (E) Cell viability quantification was determined by flow cytometry analysis of annexin V and propidium iodide (PI)‐stained NB‐4, HL‐60 or KG‐1 cells untreated or treated with 2‐DG instead of glucose, for 24 h. The results presented as mean ± SEM of, at least, 3 independent biological replicates. One‐way ANOVA and Tukey's post hoc test were used to compare the extracellular glucose and lactate levels, the [Lactate]/[Glucose] ratio and the intracellular ATP levels between NB‐4, HL60 and KG‐1 cells. Annexin V/PI data were analysed using 2‐way ANOVA and Bonferroni's post hoc test. *P < .05; **P < .01; ***P < .001

Metabolic reprogramming, the switch from oxidative to glycolytic metabolism, ensures a rapid production of ATP and biosynthetic precursors that confer adaptive advantages and long‐term maintenance to cancer cells.^10^, ^35^ Although enhanced glucose metabolism was recently described in AML,^12^, ^13^ the association between the altered energetic metabolism and the pathogenesis of different AML cells remains largely unclear. Results herein presented showed that not all AML cells prefer a glycolytic metabolism, as revealed by the quantification of extracellular glucose and lactate levels. NB‐4 cells showed lower extracellular glucose levels associated with higher extracellular lactate concentration when compared to HL‐60 and KG‐1 cells (Figure 1A,B) indicating a higher glucose consumption and lactate production. In contrast, KG‐1 cells exhibited lower glucose uptake and lactate release in comparison with NB‐4 and HL‐60 cells (Figure 1A,B). Thus, the tested AML cells can be sorted from a preferential glycolytic metabolism, displayed by NB‐4 cells, to a high oxidative metabolism, presented by KG‐1 cells, assuming HL‐60 cells an intermediate position. This AML glycolytic dependence is also supported by the ratio of [Lactate]/[Glucose], which was clearly higher for NB‐4 cells followed by HL‐60 and KG‐1 cells (Figure 1C). Consistently, data showed NB‐4 cells as those exhibiting the highest intracellular ATP levels followed by HL‐60 and KG‐1 cells (Figure 1D), in agreement with the production of ATP by time unit as result of their glycolytic rates (Figure 1A‐C). To further confirm the distinct energetic metabolism, AML cells were exposed to 2‐deoxy‐d‐glucose (2‐DG), a synthetic glucose analogue that cannot undergo glycolysis.^36^ Results demonstrated a drastic reduction in NB‐4 and HL‐60 cell's viability with no major impact on the survival of KG‐1 cells, as revealed by the annexin V/PI assay (Figure 1E). This high sensitivity of NB‐4 and HL‐60 cells to the glycolytic inhibitor 2‐DG further supports the glycolytic requirements of these cells (Figure 1A‐C). Overall, these results showed that the tested AML cells display a distinct energetic metabolism, with NB‐4 and HL‐60 cells being highly dependent on the glycolytic metabolism while KG‐1 cells appear to be more dependent on OXPHOS metabolism.

### 3.2 Complexity of the mTORC1 activation network and autophagy regulation in AML cells

**Figure 2:**
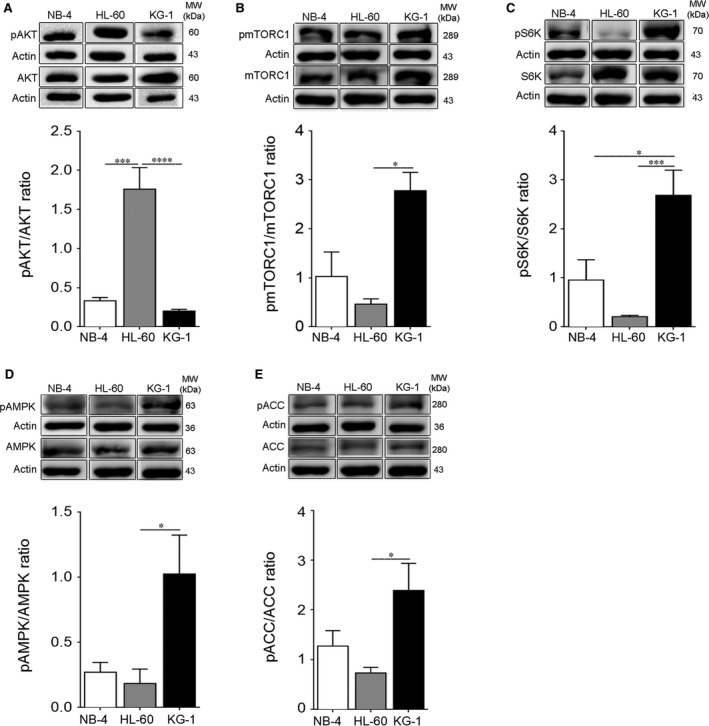
NB‐4 and HL‐60 cells exhibit AKT activation while KG‐1 cells display a constitutive AMPK and mTORC1 co‐activation. NB‐4, HL‐60 and KG‐1 cells were maintained for 24 h in normal growth medium. (A) Activation of AKT was determined by immunoblotting analysis of phosphorylated (Ser473) AKT levels. Activation of (B) mTORC1 and (C) S6K was also assessed by immunoblotting analysis of phosphorylated (Ser2448) mTORC1 and phosphorylated (Thr389) S6K levels, respectively. Activation of (D) AMPK and (E) ACC was evaluated by immunoblotting analysis of phosphorylated (Thr172) AMPK and phosphorylated (Ser79) ACC levels, respectively. Actin was used as loading control. Densitometric analysis was performed, and bands were quantified using the ImageLab4.1™ software. The results presented as mean ± SEM of, at least, 3 independent biological replicates. One‐way ANOVA and Tukey's post hoc test were used to compare the densitometric analysis of pAKT/AKT, pmTORC1/mTORC1, pS6K/S6K, pAMPK/AMPK and pACC/ACC ratios between NB‐4, HL‐60 and KG‐1 cells. *P < .05; ***P < .001

The reprogramming of energetic metabolism in tumour cells is mainly driven by the deregulation of the nutrient‐sensing pathways.^14^ The occurrence of mTORC1 constitutive activation independent of PI3K/AKT and the additional possibility of AMPK activation illustrates the complexity of the interactions between the nutrient‐sensing pathways in the AML context (reviewed in Ref^15^, ^37^). To explore the crosstalk between the observed energetic metabolism and the activation pattern of the nutrient‐sensing pathways, the activation of AKT, mTORC1 and AMPK was evaluated in AML cells. Immunoblotting analysis demonstrated a clear AKT activation in NB‐4 and HL‐60 cells, as reflected by the elevated levels of phosphorylated AKT (Figure 2A). In contrast, KG‐1 cells displayed an evident constitutive co‐activation of AMPK and mTORC1, as noticed by the highest phosphorylated levels of AMPK and acetyl‐CoA carboxylase (ACC), an AMPK direct downstream target,^38^ and the increased phosphorylated levels of mTORC1 and S6K, a direct mTORC1 downstream target^39^ (Figure 2B‐E). Data concerning AMPK activation agreed with the detected intracellular ATP levels (Figure 1D), as AMPK activation occurs in the context of energy stress (high AMP/ATP ratio)^40^ and KG‐1 cells were those displaying the lowest intracellular ATP levels (Figure 1D). Knowing that AMPK may directly inhibit mTORC1 activity,^41^ the concomitant AMPK and mTORC1 activation appears to indicate a dissociative AMPK‐mTORC1 axis in KG‐1 cells.

**Figure 3:**
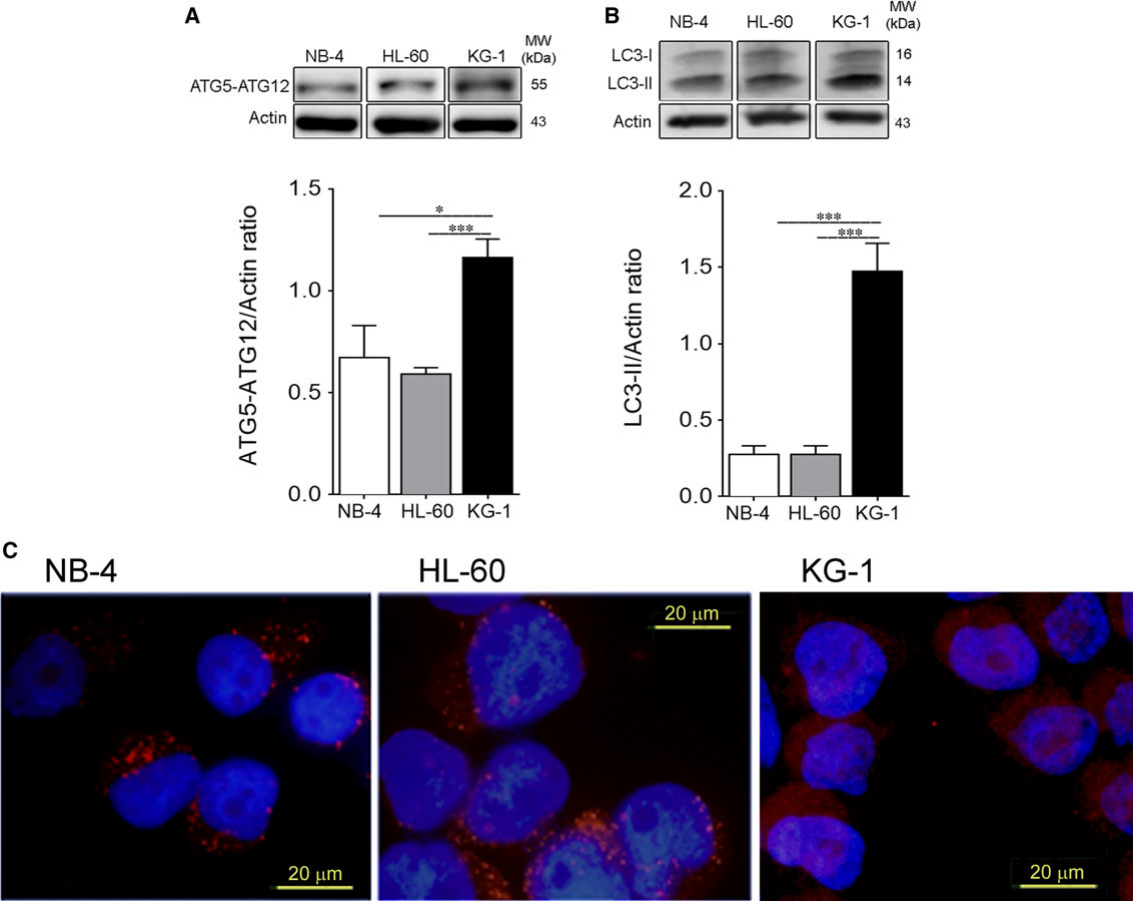
NB‐4 and HL‐60 cells show decreased autophagy flux when compared to KG‐1 cells. Autophagy flux was assessed by immunoblotting analysis of (A) Atg5‐Atg12 complex and (B) LC3 processing (I and II; all samples were incubated for 2 h with bafilomycin A1 [10 nmol/L] before the end of the experiment to block autophagy flux and to allow LC3‐II accumulation) of NB‐4, HL‐60 and KG‐1 cells incubated for 24 h in normal growth medium. Actin was used as loading control. Densitometric analysis was performed, and bands were quantified using the ImageLab4.1™ software. The results presented as mean ± SEM of, at least, 3 independent biological replicates. One‐way ANOVA and Tukey's post hoc test were used to compare densitometric analysis of Atg5‐Atg12/Actin, LC3‐II/Actin and p62/Actin ratios between NB‐4, HL60 and KG‐1 cells. *P < .05; **P < .01; ***P < .001. (C) LC3 A/B‐I/II puncta levels of AML cells were also assessed by immunofluorescence assay upon 24 h in normal growth medium. NB‐4, HL‐60 and KG‐1 cells were staining with goat LC3 anti‐Rabbit IgG antibody (red fluorescence) and samples were counter‐stained with the DNA dye DAPI (blue fluorescence). Representative images of immunofluorescence assay are presented. Bar = 20 μm

The orchestrated metabolic network perpetuated by the nutrient‐sensing pathways converges on the control of cellular catabolic processes required to maintain cellular homeostasis, such as autophagy.^42^ Given the central, although controversial, role of autophagy in the AML pathogenesis,^33^, ^43^ it is critical to comprehend not only its regulation but also its crosstalk with the metabolic signals. Therefore, autophagy was evaluated in the AML cells by immunoblotting analysis of the Atg5‐Atg12 complex, LC3 processing (I and II) and LC3 puncta.^44^ KG‐1 cells presented the highest Atg5‐Atg12 complex protein levels (Figure 3A) associated with the highest autophagy flux, as reflected by the LC3 processing (Figure 3B). Immunoblotting results of the LC3 processing were corroborated by the immunostaining of LC3 showing higher number of LC3 puncta in KG‐1 cells than in NB‐4 and HL‐60 cells (Figure 3C), which strengthens our hypothesis that autophagy is up‐regulated in KG‐1 cells.

Overall, the data herein presented showed a mTORC1 activation in all tested AML cells, whereas AKT activation was mainly observed in NB‐4 and HL‐60 cells. Importantly, our data strongly suggest that AMPK and mTORC1 are constitutively activated in KG‐1 cells. This distinct nutrient‐sensing pathway activation profile is associated with an up‐regulation of autophagy in KG‐1 cells independently of mTORC1.

### 3.3 Manipulation of nutrient‐sensing pathways impacts on autophagy and energetic metabolism of AML cells

**Figure 4:**
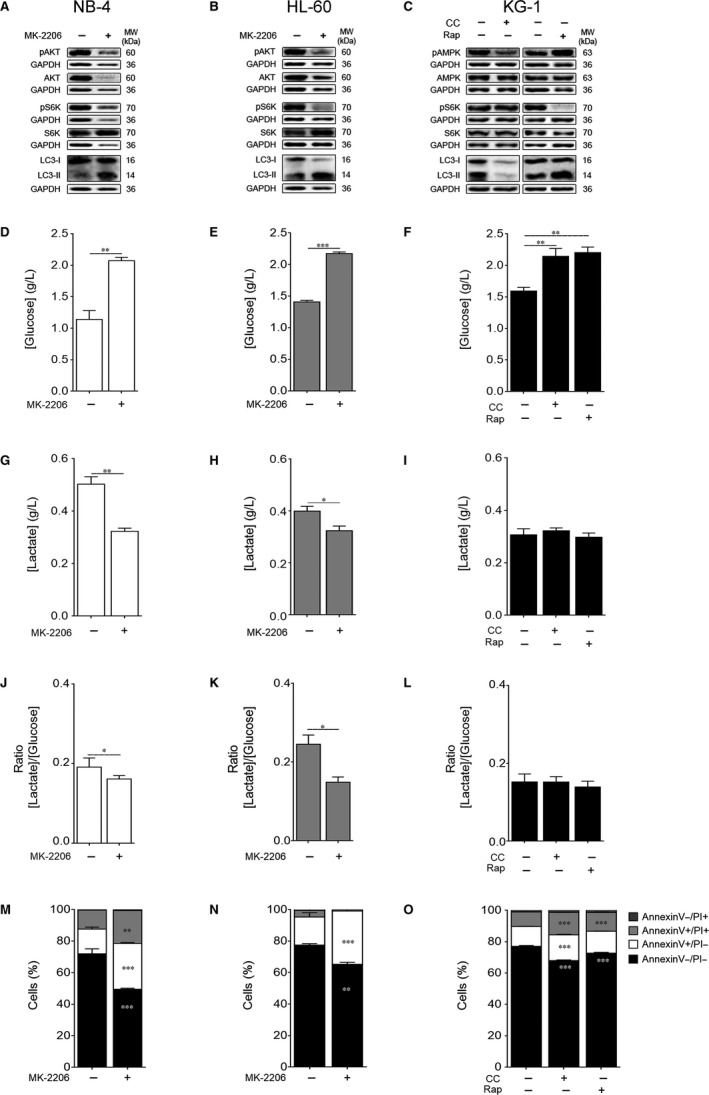
Autophagy and energetic metabolism are mainly regulated by AKT‐mTORC1 axis in NB‐4 and HL‐60 cells and by AMPK in KG‐1 cells. NB‐4 and HL‐60 cells were maintained for 24 h with or without MK‐2206 20 μmol/L while KG‐1 cells were cultured for 24 h with or without compound C (CC) 2.5 μmol/L or rapamycin (Rap) 2 μmol/L. (A‐C) Activation of AMPK, AKT and S6K as well as autophagy flux were assessed by immunoblotting analysis. Activation of AMPK and AKT was evaluated by immunoblotting analysis of phosphorylated (Thr172) AMPK and phosphorylated (Ser473) AKT levels, respectively. Activation of S6K was also evaluated by immunoblotting analysis of phosphorylated (Thr389) S6K levels. Autophagy flux was assessed by immunoblotting analysis of LC3 processing (I and II; all samples were incubated for 2 h with bafilomycin A1 [10 nmol/L] before the end of the experiment to block autophagy flux and to allow LC3‐II accumulation). GAPDH was used as loading control. The results are representative of, at least, 3 independent biological replicates. (D‐F) Extracellular glucose and (G‐I) lactate levels were determined using glucose and lactate enzymatic detection kits. (J‐L) Ratio between the extracellular lactate and glucose levels ([Lactate]/[Glucose]). The results presented as mean ± SEM of, at least, 3 independent biological replicates. Student's *t* test was applied to compare the extracellular glucose and lactate levels as well as the [Lactate]/[Glucose] ratio between untreated and MK‐2206‐treated NB‐4 and HL‐60 cells. One‐way ANOVA and Tukey's post hoc test were used to compare the extracellular glucose and lactate levels as well as the [Lactate]/[Glucose] ratio between untreated and CC‐ or Rap‐treated KG‐1 cells. *P < .05; **P < .01; ***P < .001. (M‐O) Cell viability quantification was determined by flow cytometry analysis of annexin V and PI‐stained NB‐4, HL‐60 or KG‐1 cells. The results presented as mean ± SEM of, at least, 3 independent biological replicates. Annexin V/PI data were analysed using the 2‐way ANOVA and Bonferroni's post hoc test. **P < .01; ***P < .001

Data described above suggest an AKT activation associated with a decreased autophagy flux in NB‐4 and HL‐60 cells, pointing to AKT/mTORC1 as the major regulator of autophagy in these cells. To better understand this metabolic coordination and regulation, NB‐4 and HL‐60 cells were treated with the AKT inhibitor MK‐2206. As expected, MK‐2206 promoted a clear reduction of AKT and mTORC1 (assessed by the S6K phosphorylated levels) activation with a concomitant increase of autophagy flux (Figure 4A,B), ascribing to the AKT‐mTORC1 axis a key role on the autophagy regulation of NB‐4 and HL‐60 cells. On the other hand, to decipher the relevance of the AMPK‐mTORC1 axis in KG‐1 cells, an AMPK inhibitor, compound C (CC), or a mTORC1 inhibitor, rapamycin (Rap), were used. Results presented in Figure 4C showed that CC promoted a reduction in the AMPK activation with no major impact on the mTORC1 activity (detected by the S6K phosphorylated levels), which was accompanied by a significant decline on the autophagy flux (Figure 4C). These data favour, once again, a dissociation of the AMPK‐mTORC1 axis and reinforce AMPK as the major regulator of autophagy in KG‐1 cells. As expected, Rap treatment induced a clear inhibition of mTORC1 activity with a further increase of autophagy flux (Figure 4C), suggesting that although AMPK is the main autophagy regulator in KG‐1 cells, mTORC1 is still able, to some extent, to negatively control autophagy. Overall, results point to a similar metabolic profile of NB‐4 and HL‐60 cells indicating the AKT‐mTORC1 axis as the major negative regulator of autophagy in these cells, in contrast to KG‐1 cells, in which autophagy is positively controlled by AMPK.

The impact of the nutrient‐sensing pathways inhibition on the energetic metabolism of AML cells was also assessed. Inhibition of AKT by MK‐2206 resulted in a significant increase in the extracellular glucose levels associated with an evident decrease in the extracellular lactate concentration of NB‐4 and HL‐60 cells (Figure 4D,E,G,H), suggesting a decrease in the glucose consumption and lactate production of these cells. Indeed, the observed decreased glycolytic metabolism promoted by MK‐2206 in the NB‐4 and HL‐60 cells was confirmed by the [Lactate]/[Glucose] ratio (Figure 4J,K). These results point to AKT as a main player on the regulation of the glycolytic metabolism of NB‐4 and HL‐60 cells. Treatment of KG‐1 cells with CC or Rap resulted in increased extracellular glucose levels and no major alterations in the extracellular lactate concentration (Figure 4F,I,L). The maintenance of lactate concentration with decreased glucose consumption suggests a glucose‐independent source of lactate and is compatible with the predominant OXPHOS metabolism displayed by these cells (Figure 1).

In summary, the results obtained in this study indicate the fundamental role of AKT in controlling glycolysis of both NB‐4 and HL‐60 cells while supporting the low relevance of glycolysis in the KG‐1 cells’ metabolism. Furthermore, the results herein presented show, for the first time, a relation ship between energetic metabolism and autophagy, both controlled by nutrient‐sensing pathways.

### 3.4 Targeting nutrient‐sensing pathways sensitizes NB‐4 and HL‐60 but has a minor impact on KG‐1 cells

The impact of manipulating AKT, mTORC1 and AMPK on the survival of AML cells is still controversial.^18^, ^20^, ^21^, ^43^, ^45^ Knowing that inhibition of these nutrient‐sensing pathways has a major impact on autophagy and energetic metabolism of AML cells, the viability of these cells was evaluated. Data showed a significant decrease on the viability of NB‐4 (Figure 4M) and HL‐60 (Figure 4N) cells upon exposure to MK‐2206, pointing to AKT as critical for the survival of both types of AML cells. Given that AKT inhibition resulted in an increase of autophagy flux in both NB‐4 and HL‐60 cells (Figure 4A,B), the MK‐2206‐promoted cell death is associated with autophagy, implicating autophagy as an anti‐tumoural process in NB‐4 and HL‐60 cells. Treatment of KG‐1 cells with CC or Rap resulted in a modest, although significant, decrease in their viability (Figure 4O). Together with the distinct effects that these compounds had on autophagy flux (Figure 4C) and with the independency of glycolysis (Figure 1), AMPK and mTORC1 do not seem to be an attractive target for KG‐1 cells. Most probably this phenomenon reflects the conflicting metabolic signals resulting in the constitutive co‐activation of AMPK and mTORC1.

## 4 DISCUSSION

The genetic and epigenetic heterogeneity, compromising differentiation, proliferation and self‐renewal of hematopoietic stem cells and myeloid progenitors, is a fundamental property of AML. This multitude of AML scenarios not only has hampering the understanding of AML's pathogenesis and classification but also the development of efficient therapeutic approaches. Different studies have been trying to establish a metabolic signature of AML cells^12^, ^13^, ^46^, ^47^; however, the heterogeneous nature of this group of disorders has been responsible for the current controversial literature either using cell line models or patient's derived samples, as primary cells or serum. Herein, we used 3 different AML cell line models, namely the KG‐1 leukaemia cell line derived from a patient with erythroleukaemia, NB‐4 a model of acute promyelocytic leukaemia (patient in second relapse) and HL‐60 a M2‐derived cell line. These cell lines were chosen because they are very well characterized and widely used as representative of different AML subtypes. Using the 3 different AML cell line models, we highlight the multitude of metabolic cellular scenarios that might arise even in very closely related cell lines such as NB‐4 and HL‐60 cells that belong to a different genetic cluster when compared to KG‐1 cells.^48^

Regarding the energetic metabolism, our results categorize the different AML cells with NB‐4 as a glycolytic cell line, as reported in some studies,^49^, ^50^, ^51^ HL‐60 cells mainly dependent on glycolysis^12^ and KG‐1 cells displaying a predominant OXPHOS metabolism. These results not only showed that 2 closely related cell lines, NB‐4 and HL‐60, present different energetic metabolism, but also that KG‐1 cells are mainly OXPHOS dependent, as reflected by the carbon flux through mitochondria for lactate production (Figure 1). These data suggest that there is no specific metabolic profile associated with AML tumorigenesis and that different metabolic frames can sustain AML cells’ survival and proliferation.

Altered metabolism is a direct response to growth factor signalling and to nutrient‐sensing pathways such as AMPK, mTORC1 and PI3K/AKT. The data herein presented also show a correlation between a predominant glycolytic metabolism of AML cells and activation of AKT in NB‐4 and HL‐60 cells, in agreement with previous observations showing that AKT promotes glycolysis in U937 AML cells.^19^ Furthermore, the AKT activation exhibited by NB‐4 and HL‐60 cells (Figure 2A) is associated with reduced autophagy flux, indicating that PI3K/AKT/mTORC1 activation seems to drive anabolic metabolism and tumorigenesis in certain AML scenarios by impacting on autophagy.

KG‐1 cells displayed a completely distinct metabolic profile with a major dependency of OXPHOS metabolism and carbon flux through Krebs cycle. Remarkable, in KG‐1 cells, a constitutive co‐activation of AMPK and mTORC1, often perceived as antagonists, is observed. The opposite role of AMPK and mTORC1 on metabolic reprogramming is supported by studies showing that AMPK is an inducer of OXPHOS in T cell acute lymphoblastic leukaemia^52^ and mTORC1 a promoter of the glycolytic metabolism in several AML cell lines and human primary AML samples.^18^ Accordingly, our data point to AMPK as the potential responsible for the increased oxidative metabolism exhibited by KG‐1 cells (Figure 1) imposing its action to mTORC1. A similar contradictory metabolic scenario was already reported in myoblasts in response to amino acids.^53^ The authors proposed that the concurrent activation of AMPK and mTORC1 is implicated in the maintenance of protein homoeostasis and on the fuel of metabolites for biosynthetic processes.^53^ The relevance of amino acid signalling and mTORC1 PI3K/AKT‐independent activation in the context of AML remains to be explored. To the best of our knowledge, this is the first time that the mechanism of AMPK and mTORC1 constitutive co‐activation is described in KG‐1 cells, which deserves future exploration in the regulation of AML cells metabolism.

The novel observation of a constitutive co‐activation of AMPK and mTORC1 in KG‐1 cells (Figure 2B‐E) associated with increased autophagy (Figure 3) is striking. Knowing that AMPK can induce autophagy in mTORC1‐independent pathways^27^, ^28^, ^29^ and that mTORC1 is a negative regulator of autophagy,^29^ the obtained data suggest a dissociative AMPK‐mTORC1 axis with AMPK sustaining autophagy in KG‐1 cells. In fact, the reports of Sujobert et al and Pezze et al also showed a direct induction of autophagy by AMPK even in the presence of mTORC1 activation.^21^, ^53^

Using human AML bone marrow mononuclear cells and an AML mouse model, Watson et al^54^ showed that autophagy limits glycolytic metabolism in the AML context. Our data show that increased glycolytic dependence is associated with a reduced autophagy flux (Figure 3), while a diminished glycolytic metabolism, as observed in KG‐1 cells (Figure 1), was accompanied by elevated autophagy flux (Figure 3). These findings highlight the contribution of autophagy in the regulation of energetic metabolism, indicating an autophagy role in the control of the glycolytic metabolism of the different tested AML cells. Our findings suggesting that activated AMPK‐autophagy axis is responsible for the augmented oxidative metabolism displayed by KG‐1 cells are in agreement with data obtained in T‐ALL cells and with mixed lineage AML model.^52^, ^54^ Furthermore, these studies suggest that the complex nutrient‐sensing network regulating autophagy can have a major impact on AML pathogenesis and response to therapy, including combined therapy with inhibitors of mTOR.

Overall the data presented on the inhibition of nutrient‐sensing pathways and its impact on the AML cells’ survival demonstrate that targeting nutrient‐sensing pathways sensitizes NB‐4 and HL‐60 cells to chemotherapy but has a minor impact on KG‐1 cells survival, which emphasizes the idea that nutrient‐sensing pathways may not constitute a promising and effective therapeutic target.

In the present study, our results show that different AML cells have different energetic, metabolic and autophagy patterns that are tightly interconnected in the regulation of AML cells’ survival. Our data also point to AKT as the major regulator of energetic metabolism and autophagy in NB‐4 and HL‐60 cells. In KG‐1 cells, the energetic metabolism and autophagy seem to be regulated by AMPK and mTORC1. These results highlight that the genetically, metabolically and clinically heterogeneity of AML should be considered and might justify the general modest growth‐inhibitory effects in preclinical AML models and clinical trials of mTOR inhibition.^25^, ^55^ Furthermore, the results highlight the relevance that comparative studies implying AML cell lines have on the determination of the anti‐leukaemia efficacy, particularly, of the effectiveness of combinatory therapy with conventional and new targeted agents. The therapeutic approach to AML diseases must pass through personalized therapy adapted to the heterogeneity of this group of neoplasms.

## CONFLICT OF INTEREST

The authors declare that they have no conflict of interests concerning the contents of this article.

## AUTHOR CONTRIBUTION

PL and IC designed the research study; BSM and OP analysed the data; PL, BSM and OP wrote the manuscript; OP, BSM and AT performed the research; IC and HG performed a critical revision of the manuscript. All authors have read and approved the manuscript.
